# The composite quality score for the appraisal of prospective controlled clinical therapy trials in systematic reviews and its limits

**DOI:** 10.3389/fmed.2023.1201951

**Published:** 2023-06-28

**Authors:** Steffen Mickenautsch, Stefan Rupf, Veerasamy Yengopal

**Affiliations:** ^1^Faculty of Dentistry, University of the Western Cape, Cape Town, South Africa; ^2^Department of Community Dentistry, School of Oral Health Sciences, Faculty of Health Sciences, University of the Witwatersrand, Johannesburg, South Africa; ^3^Review Centre for Health Science Research, Johannesburg, South Africa; ^4^Synoptic Dentistry, Saarland University, Saarbrücken, Germany

**Keywords:** composite quality score, clincial rial, trial appraisal, systematic review, bias

## Abstract

Systematic reviews of prospective controlled clinical therapy trials are one of the most important sources of information in modern medicine. Besides the systematic search for and statistical pooling of current clinical trial data for a particular type of therapy, systematic reviews also have the task of appraising the quality of trial results. The quality of trial results may be diminished by low internal trial validity, due to systematic error (bias). A high risk of bias may likely cause the reported trial results to be diverted from the actual true therapeutic effect and thus render it unsuitable for clinical guidance. According to the Cochrane Collaboration, the risk of bias in clinical therapy trials should be assessed using its Risk of Bias tool, Version 2 (RoB 2). However, the tool has been established to have poor inter-rater reliability, with a limited empirical evidence base and described as complex and demanding. Against this background, the composite quality score (CQS) has been developed as a possible alternative trial appraisal tool, characterised by high epistemic rigour, empirical evidence base, inter-rater reliability and ease of use. This article presents the current evidence of the CQS and its limitations.

## 1. Introduction

According to the Cochrane Collaboration, the risk of bias in clinical therapy trials should be assessed using its Risk of Bias tool, Version 2 (RoB 2). The tool consists of 22 signalling questions to five bias domains (randomisation process, deviation from intended interventions, missing outcome data, measurement of outcome, and selection of the reported result) (1).

The tool was developed between 2015–2019, based on a lengthy process of expert consensus, repeated cycles of discussion, piloting and rewording. The development was further supported by the results of a systematic review of meta-epidemiological studies concerning empirical evidence of bias effect on clinical trial results (2), a systematic review of types of bias in epidemiology (1) and a cross-sectional study on selective outcome reporting (3).

Despite its rigorous development process, the RoB 2 tool has to date been shown to have poor inter-rater reliability (IRR: Fleiss’ Kappa 0.16; 95% CI: 0.08–0.24) and its application has been described as complex and demanding, therefore requiring intensive formal training and the conduct of pilot runs before it may correctly be applied (4). Even after the inclusion of an intensive rater-calibration process, including a total of 40 h over a period of 3 months, the overall IRR remained moderate (IRR = 0.42) only (5).

During its developmental process, the RoB 2 tool is claimed to have been based on empirical evidence from meta-epidemiological studies (2). However, the systematic review by Page et al. (2) could provide evidence in support of only 5 out of the 22 signalling questions of the tool. This evidence supports the signalling question number 1.1 and 1.2, namely whether the allocation concealment was random (7% overestimation of trials with inadequate or unclear randomisation: ROR 0.93, 95% CI: 0.86–0.99, *I*^2^ = 0%) and whether the allocation sequence was concealed (10% overestimation of trials with inadequate or unclear allocation concealment: ROR 0.90, 95% CI: 0.84–0.97, *I*^2^ = 28%), respectively. The evidence also supports in principle the signalling questions number 4.3–4.5 in the domain “bias in measurement of the outcome; that is, in support for blinded assessment of susceptive outcomes and double-blinding (23% overestimation of trials with inadequate or unclear double-blinding: ROR 0.77, 95% CI: 0.61–0.93). However, “blinding” was not explicitly included in formulating the tool’s questions. The systematic review did not identify empirical evidence supporting 3 out of the tool’s 5 bias domains (including 17 out of its total 22 signalling questions), namely “deviation from intended intervention,” “missing outcome data” and “selective reporting” (2).

Besides its poor inter-rater reliability (IRR) and limited evidence base, its complex and demanding application may be the reason why a low adherence to the RoB 2 tool among Cochrane protocols and systematic reviews was observed and why most Cochrane reviewers themselves choose not to use the tool (6). The complexity of applying the RoB 2 tool stands in contrast to the steadily increasing volume of clinical intervention trials worldwide (7) and thus the subsequent need arises for more timely, less complicated, yet effective and reliable trial appraisal methods.

In addition to the above, the RoB 2 tool assigns an overall “low-bias risk” status to trials that have been judged to be of “low-bias risk” in all of its five single bias domains, even though any methodological trial error that lies outside its five domains may completely invalidate the reported trial results.

Against this background, the composite quality score (CQS) has been developed as a possible alternative for the appraisal of prospective controlled clinical therapy trials in systematic reviews.

## 2. The composite quality score

The development of the CQS as a possible trial appraisal tool started in 2019 with the epistemic consideration that regardless of how many trial appraisal criteria a trial has fully complied with, the judgment of such a trial as being of “low-bias risk” cannot be justified but a single methodological error may render a trial to actually be of “high-bias risk.” Accordingly, three evidence-based criteria describing trial design requirements (random allocation sequence generation, allocation sequence concealment, minimum sample size) that are essential (albeit not sufficient) for the trial results to reflect therapeutic truth were established. The criteria were intentionally worded in a simplified and least restrictive manner, based on the rationale that the lower the stringency of an appraisal criterion, the higher the certainty of “high-bias risk” when such criterion is not met (8).

To explore the applicability of the novel CQS approach for trial appraisal, it was applied in the field of restorative dentistry. Based on a systematic literature search, 683 prospective clinical controlled trial reports were identified out of a total of 14,694 citations. Of these, the bias risk of 99.7% of trials could be appraised and identified as high, despite the low stringency of the applied criteria (9). In 2021, the inter-rater reliability of the CQS approach was investigated for the first time. The results showed a high inter-rater reliability: Brennan–Prediger coefficient (BPC) of 0.95; 95% CI: (0.87–1.00), which compared favourably to that of the first RoB tool version, which ranged from BPC –0.07; 95% CI: −0.42 to 0.28 and 0.34; 95% CI: −0.05 to 0.73. Most of the differences between the RoB and the CQS were statistically significant (*p* < 0.05) in favour of the CQS (10).

The findings of all three preliminary investigations (8–10) were summarised and presented as the first version of the CQS (CQS-1) in 2023 (11).

In order to extend the applicability and evidence base of the CQS-1 for all fields of clinical therapy, a systematic review of meta-epidemiological studies was conducted. Based on its results, one new criterion concerning double-blinding was added, and the original criteria concerning random allocation sequence generation and minimum sample size were amended (12). Consequently, all four criteria of the resulting new CQS version (CQS-2) were based on meta-epidemiological evidence, indicating statistically significant (*p* < 0.05) over- or underestimation of the true therapeutic effect estimate for trials where such criteria were not met (12). Based on additional empirical evidence, this systematic review was partially updated to improve the wording and applicability of the criterion concerning the appraisal of allocation concealment, resulting in a further refined CQS version (CQS-2B) (13).

Following the extension of the original CQS-1, the CQS-2 was assessed to establish whether its extended format extension would have negatively affected the high inter-rater reliability of the first version. The results of this study showed that CQS extension had no negative effect and that the CQS-2 version was also associated with very high inter-rater reliability (BPC 1.00; 95% CI: 0.94–1.00) and did not statistically differ significantly (*p* > 0.05) from that of the CQS-1 (BPC 0.85; 95% CI: 0.64–1.00) (14). The high inter-rater reliability was achieved despite the fact that all four raters had no extensive expertise in the conduct of systematic reviews of randomised controlled trials, nor extensive expert-content knowledge concerning the topics of the rated trials. Also, besides the provision of written information about how to apply the CQS, no calibration or training in using the CQS was carried out prior its application (14). For these reasons, the CQS can be considered less complex and less demanding, yet more reliable than the RoB 2 tool.

The result of this assessment led to the development of a CQS version (CQS-2B) that included four criteria related to the random allocation to treatment groups, concealment of such allocation, double-blinding and sample size minimum (Table 1).

**Table 1:** CQS-2B appraisal criteria.

Criterion I	“Randomisation” for allocation to treatment groups is in some form reported in the text
Criterion II	Any assurance that the patient allocation to treatment groups according to the random sequence was applied by an independent agent or agency, not otherwise involved in the trial, is in some form reported in the text
Criterion III	Double-blinding or the blinding of at least two out of the three groups: trial participants, trial personnel and trial outcome assessors in some form reported in the text
Criterion IV	The sample size of any particular treatment group reported in the trial is not less than *N* = 100

Its application comprised: (i) binary trial report rating per appraisal criterion (scores: 0 = no/invalid/falsified, 1 = yes/corroborated); (ii) multiplication of all criterion scores to an overall appraisal score, (iii) identification of invalid/falsified trial reports based on a zero overall appraisal score.

During its application, several corroboration (C-) levels are recognised. These levels indicate the number of consecutive criteria that a trial has complied with (e.g., level C3 indicates compliance with criterion I–III; level C4 indicates compliance with criterion I–IV, etc.). A corroboration level for a particular trial is reached before one criterion is rated with a 0-score or when all criteria are rated with a 1-score, for example corroboration level C3: criterion I–III = 1-score, criterion IV = 0-score; corroboration level C4: all criteria = 1-score. After a criterion has been rated with a 0-score, the C-level of a trial remains the same even if a following criterion is rated with a 1-score, for example corroboration level C1: criterion I = 1-score, criterion II = 0-score, criterion III = 1-score. No certainty of “low-bias risk” is ascribed to an overall 1-score appraisal result, which indicates only that during the appraisal process no evidence for high-bias risk could be established so far.

It was explored whether systematic review conclusions originally based on Cochrane’s second version of its Risk of Bias tool (RoB 2) would differ when the CQS-2B is used for trial appraisal instead. The results of this study provided justification for the testable hypothesis that trial appraisal using the CQS-2B provides more conservative conclusions based on similar data than trial appraisal using Cochrane’s RoB 2 tool (15).

## 3. CQS limitations

Despite its high epistemic rigour, evidence base and inter-rater reliability, the current version of the CQS has certain limitations that need to be taken into account when considering its application in systematic reviews of prospective controlled clinical therapy trials. These include the following items.

### 3.1. Verification of low-bias risk

As per the design, the CQS-2B is not a tool for verifying low-bias risk. Trials that are awarded 1-scores for all four appraisal criteria may still have serious flaws in other aspects of their methodology. This is a fully intended aspect of the CQS approach in line with the epistemic principles of deductive falsification (8).

One example is the clinical trial by Sitthisettapong et al. (16) concerning the clinical effect of 10% w/v calcium phosphopeptide-amorphous calcium phosphate (CPP-ACP) paste for 1 year when added to regular toothbrushing with fluoridated toothpaste to prevent dental caries in pre-school children. The trial reports to be “randomised” (criterion I = 1-score) with the allocation of experimental or control group were determined by an assistant who was not involved in the clinical aspects of the study (criterion II = 1-score), “double-blind” (criterion III = 1-score) and included patients of which “150 were assigned to the experimental condition and 146 to the control condition” (criterion IV = 1-score).

However, subsequent further in-depth appraisal reveals that patient allocation was neither random nor concealed but alternated with patients “with odd ID numbers assigned to the experimental treatment, and those with even ID numbers to the control treatment.” This form of alternation may cause the allocation of patients to either group to be visible, which renders the study open to a high risk of selection bias. Furthermore, the important baseline variable “falling asleep with a bottle” was statistically significantly more distributed among the experimental group (*p* = 0.008). If randomisation had been effective, then all baseline variables should have been equally distributed between the groups.

This example shows that corroborated trials according to the CQS-2B cannot be considered as low-bias risk. Instead, corroborated trials may be considered eligible for further in-depth appraisal as shown in the example above. Such appraisal may not be confined within the criteria of any specific trial appraisal tool but may need to be tailored specifically to the particularities of each trial. In contrast, a further in-depth appraisal may not be warranted for trials that have been already falsified at C1–4 levels since the appraisal process has already provided sufficient reason to consider such trials as of high-bias risk. In that way, the CQS-2B may contribute to a more timely and efficient trial appraisal, particularly of a large volume of prospective controlled clinical therapy trials (7).

### 3.2. The discrepancy between not reported versus actual trial characteristics

Not all actual trial characteristics that were performed during the conduct of a trial, required by the CQS criteria, may have been reported by trial authors. For example, trial randomisation may have included central allocation, but this was not made explicit in the published trial report. Trial appraisal using the CQS-2B would thus have assigned an erroneous 0–score to its criterion II.

Contacting trial authors during a systematic review of trials may appear to be a possible solution but can be affected by recall bias, the fact that such author responses have not been part of the original peer-review process and the reduction of possible reproducibility of the systematic review results at a later stage. Trial appraisal remains by its nature qualitative text analysis only. This is valid for any trial appraisal tool, such as the CQS-2B or Cochrane’s RoB 2. The consequence is false-negative trial appraisal results that may not be remedied.

### 3.3. The discrepancy between not performed trial methods and systematic error effect

The lack of essential trial characteristics may not always lead to systematic error. This limitation is also valid for any trial appraisal tool. Trial appraisal based on qualitative text analysis can assume whether a trial is susceptible to systematic error only when essential trial characteristics are missing. Quantitative tests that can be applied during the systematic review of clinical trials may solve this problem. Currently, there are only a few tests available for this purpose, such as the highly accurate Berger–Exner test for 3rd-order selection bias (17). However, the currently existing tools do not yet cover all bias domains and thus do not replace trial appraisal based on qualitative text analysis. Therefore, the CQS-2B can establish only a “high-bias risk” of a trial but cannot actually provide proof that a trial result is biased. For this reason and in agreement with guidelines for the RoB 2 tool (1), it is strongly recommended that systematic reviews with the CQS-2B should not exclude high-bias risk trial data but always include stratification by overall bias risk/corroboration level for trial outcomes or endpoints during meta-analysis.

## 4. Conclusion

The CQS has been developed based on high epistemic rigour, meta-epidemiological evidence and inter-rater reliability. However, like all trial appraisal tools, it has limitations that need to be considered during its application in systematic reviews of prospective controlled clinical therapy trials.

## Author contributions

SM contributed to conception and design of the review and wrote the first draft of the manuscript. SM, SR, and VY commented and improved the manuscript. All authors contributed to the article and approved the submitted version.

## Conflict of interest

The authors declare that the research was conducted in the absence of any commercial or financial relationships that could be construed as a potential conflict of interest.

## Publisher’s note

All claims expressed in this article are solely those of the authors and do not necessarily represent those of their affiliated organizations, or those of the publisher, the editors and the reviewers. Any product that may be evaluated in this article, or claim that may be made by its manufacturer, is not guaranteed or endorsed by the publisher.
